# Endogenous acrolein accumulation in *akr7a3* mutants causes microvascular dysfunction due to increased arachidonic acid metabolism

**DOI:** 10.1016/j.redox.2025.103639

**Published:** 2025-04-17

**Authors:** Xin Zhang, Johannes Gschwind, Vanessa Erben, Katrin Bennewitz, Xiaogang Li, Carsten Sticht, Gernot Poschet, Ingrid Hausser, Thomas Fleming, Julia Szendroedi, Peter Paul Nawroth, Jens Kroll

**Affiliations:** a Department of Vascular Biology, ECAS, Medical Faculty Mannheim, Heidelberg University, Mannheim, 68167, Germany; b NGS Core Facility, Medical Faculty Mannheim, Heidelberg University, Mannheim, 68167, Germany; c Metabolomics Core Technology Platform, Centre for Organismal Studies, Heidelberg University, Heidelberg, 69120, Germany; d Institute of Pathology IPH, EM Lab, Heidelberg University Hospital, Heidelberg, 69120, Germany; e Department of Internal Medicine I and Clinical Chemistry, Heidelberg University Hospital, Heidelberg, 69120, Germany; f Medical Clinic and Polyclinic II, University Hospital Dresden, Dresden, 01307, Germany

**Keywords:** Acrolein, Arachidonic acid metabolism, Aldo-keto reductase, Ocular vascular diseases, Kidney alteration, Zebrafish

## Abstract

Acrolein (ACR) is an endogenous reactive unsaturated aldehyde that can be detoxified by the aldo-keto reductase (AKR) enzyme system. While it has been shown that accumulation of ACR is associated with several health problems, including inflammation, oxidative stress, and cardiovascular disease the study aimed to analyze whether an endogenous accumulation of ACR is causal for vascular dysfunction in an *akr7a3* mutant zebrafish model. Enlargement of the hyaloid and retinal vasculature, as well as alterations in the larval pronephros and thickening of the glomerular basement membrane in the adult kidney were found upon ACR accumulation. Transcriptomic and metabolomic analyses, followed by functional validation, revealed that the up-regulation of genes controlling the arachidonic acid metabolism and activation of the leukotriene pathway are responsible for the observed microvascular changes. In conclusion, the data have identified an intrinsic function of ACR in *akr7a3* mutants that activates the arachidonic acid metabolism and subsequently disrupts vascular integrity by promoting an inflammatory response. Thus, ACR is causal in the development of vascular disease.

## Highlights

• Acrolein accumulates in *akr7a3* zebrafish mutants
• Acrolein is causal for microvascular dysfunction
• Acrolein activates the arachidonic acid metabolism

## Graphical abstract

**Figure:**
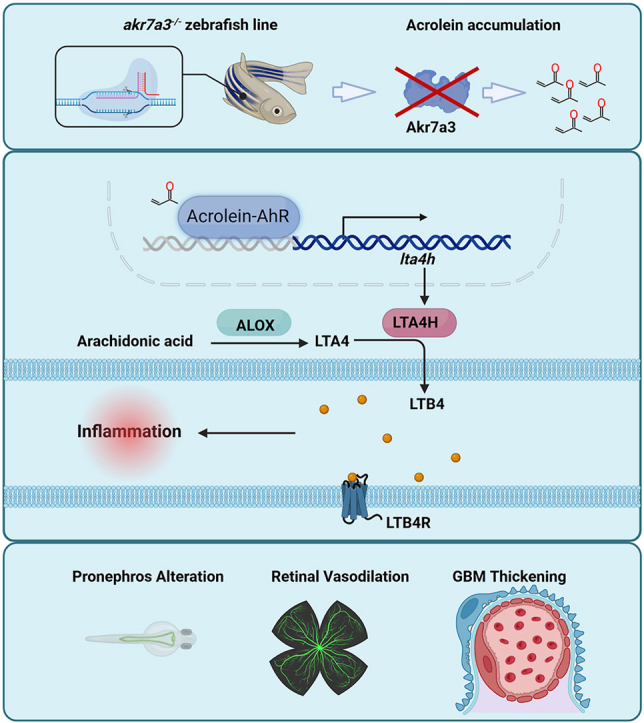

## 1 Introduction

Reactive carbonyl species (RCS) are a group of highly reactive metabolites containing one or more carbonyl groups. These RCS can be formed as by-products during the oxidation of carbohydrates, lipids, and amino acids *in vivo*. Due to their high reactivity, RCS can modify cellular macromolecules such as proteins, lipids, and DNA, leading to organ dysfunction, apoptosis, and inflammation. The accumulation of RCS is considered to be a detrimental factor in the pathogenesis of degenerative diseases such as diabetes mellitus (DM) [1], cardiovascular disease (CVD) [2], and Alzheimer's disease [3]. Therefore, normal life is only possible when formation and detoxification of these metabolites is carefully controlled.

As a member of the RCS, acrolein (ACR) is a highly reactive α,β-unsaturated aldehyde that can be derived from lipid peroxidation, polyamine metabolism, and myeloperoxidase synthesis [4,5]. ACR can easily diffuses across lipid membranes and enter various cell types. More importantly, its oxidative carbonyl group allows it to react with proteins, lipids, and DNA via either Schiff base or Michael addition reactions, leading to molecular dysfunction and cellular maladaptation [6]. Endogenous ACR production can be increased under conditions such as hyperglycemia [7], ischemia [8], and inflammation [9], highlighting its important role in the pathogenesis of various diseases. Several studies have also shown that ACR plays a pivotal role in the pathogenesis of atherosclerosis [10], diabetic retinopathy [11], cerebral vascular disease [12], and acute kidney injury [8]. Exposure to ACR can induce ferroptosis by activating endoplasmic reticulum stress [13,14] and impair insulin signaling pathways by inhibiting mitochondrial respiration [15]. In addition, ACR can promote the progression of atherosclerosis, where ACR-induced ERK phosphorylation enhances leukotriene production and matrix metalloproteinase (MMP) expression, leading to atherosclerotic lesions [16].

The above studies have used either exogenous, unphysiological ACR to induce cellular changes or metabolic disorders that increase ACR in order to study the pathogenic effects of ACR. In the absence of specific acrolein inhibitors, it remained unknown whether endogenous ACR could actually directly induce disease-like organ changes. Furthermore, the main targets of endogenously elevated acrolein have not been investigated, but vascular and renal changes are likely to be the main targets of elevated acrolein. It would therefore be essential to develop an experimental strategy to increase ACR endogenously, without external manipulation or metabolic and disease activation.

ACR can be detoxified by aldo-keto reductase (AKR) enzymes. AKRs function as NADPH-dependent oxidoreductases that catalyze the reduction of aldehydes and ketones to their corresponding primary and secondary alcohols, respectively. Our previous study showed that the loss of Akr1a1a in zebrafish resulted in ACR-mediated impaired glucose homeostasis and subsequent activated angiogenic retina and a glomerular basement membrane thickening [17]. Additional studies have also identified a role for Akr7a1 and Akr7a2 in mitigating cytotoxicity and tissue damage by protecting against ACR-induced DNA damage *in vivo* and in vitro [18,19]. Taken together, these studies identified ACR as a substrate of the AKR superfamily and revealed its crucial role in regulating metabolic homeostasis and cellular function. Akr7a3 is a member of the AKR superfamily and shares similar crystal structures and protein functional domains with other AKRs, but its substrate specificity and biological function are largely unknown. It has been suggested that Akr7a3 is responsible for the detoxification of aflatoxin B1 by reducing protein adducts, thereby inhibiting AFB 1-induced cytotoxicity [20]. In addition, Akr7a3 may play a role in protecting against acetaminophen (APAP)-induced hepatotoxicity [21] and contributes to hormone metabolism, and carcinogen degradation [22].However, whether ACR accumulates in *akr7a3*^*−/−*^ mutants and whether endogenously increased ACR regulates endothelial and renal dysfunction remained unknown.

Therefore, the study was designed to address the following questions. Does ACR accumulate in *akr7a3*^*−/−*^ mutants? What are the consequences of endogenous ACR accumulation on the vasculature and kidney in *akr7a3*^*−/−*^ zebrafish and what is the molecular mechanism of ACR activity? Our data showed that endogenous ACR accumulation in *akr7a3*^*−/−*^ mutants activates the arachidonic acid metabolism, leading to increased vessel diameter in the retina and thickening of the glomerular basement membrane (GBM) in the kidney. ACR has therefore been identified as a causal factor in the development of microvascular disease.

## 2 Results

### 2.1 Generation and validation of *akr7a3*^*−/−*^*mutant* zebrafish

Previous studies have shown that the AKR superfamily plays a crucial role in detoxifying reactive aldehydes and maintaining glucose metabolism homeostasis [17,23,24]. Among its members, *akr7a3* is conserved across species including humans and zebrafish (Figs. S1A and B). In humans, *akr7a3* was predominantly expressed in the pancreas, gastrointestinal tract, and liver and gallbladder (Fig. S1A) [[25], [26], [27]]. Multiple sequence alignment revealed that rat, human and zebrafish homologues of the Akr7a3 enzyme share a conserved active site and three substrate binding sites (Fig. S1C). Further RT-qPCR analysis revealed that *akr7a3* was predominantly expressed in the liver, with lower expression observed in the kidney and spleen in adult *akr7a3*^+/+^ zebrafish (Fig. S1D). This suggested that zebrafish could be used to study the *akr7a3* gene due to its many advantages in metabolic disease research [28,29].

To investigate the detoxification of reactive metabolites and the specific function of Akr7a3 in the metabolism and vasculature, an *akr7a3*^*−/−*^ zebrafish line was generated using CRISPR/Cas9 technology. The CRISPR-guideRNA (gRNA) was synthesized targeting exon 1 of *akr7a3* (Fig. S2A). The gRNA was injected into *Tg(fli1:EGFP)* zebrafish embryos along with Cas9 mRNA for further breeding. Subsequent validation showed that a 10 bp insertion of the *akr7a3* gene was identified by genomic DNA sequencing, resulting in a frameshift mutation in the *akr7a3* gene and a premature stop codon in the process of Akr7a3 translation (Figs. S2A and B). Subsequent experiments were performed at the DNA, RNA, and protein levels to validate the generation of an *akr7a3*^*−/−*^ mutant. The genomic DNA bands of *akr7a3*^+/+^, *akr7a3*^*−/−*^, and *akr7a3*^+/−^ could be distinguished after PCR amplification and gel electrophoresis (Fig. S2C). RT-qPCR analysis validated that *akr7a3* mRNA was significantly decreased in the liver of adult *akr7a3*^*−/−*^ zebrafish (Fig. S2D). Western blot analysis showed that the signal of Akr7a3 protein was completely lost in the eyes of adult *akr7a3*^*−/−*^ zebrafish (Fig. S2E). Furthermore, the genotype distribution of the offspring of *akr7a3*^+/−^ zebrafish is consistent with Mendelian inheritance (Fig. S2F). Interestingly, no compensation of expression in *akr7a3*^*−/−*^ mutants for *akr1a1a* and vice versa could be detected (Figs. S2G and H). In conclusion, these data indicated that the *akr7a3*^−/−^ zebrafish has been successfully established.

### 2.2 Increased acrolein in *akr7a3*^*−/−*^ zebrafish larvae and in adult organs

**Fig. 1:**
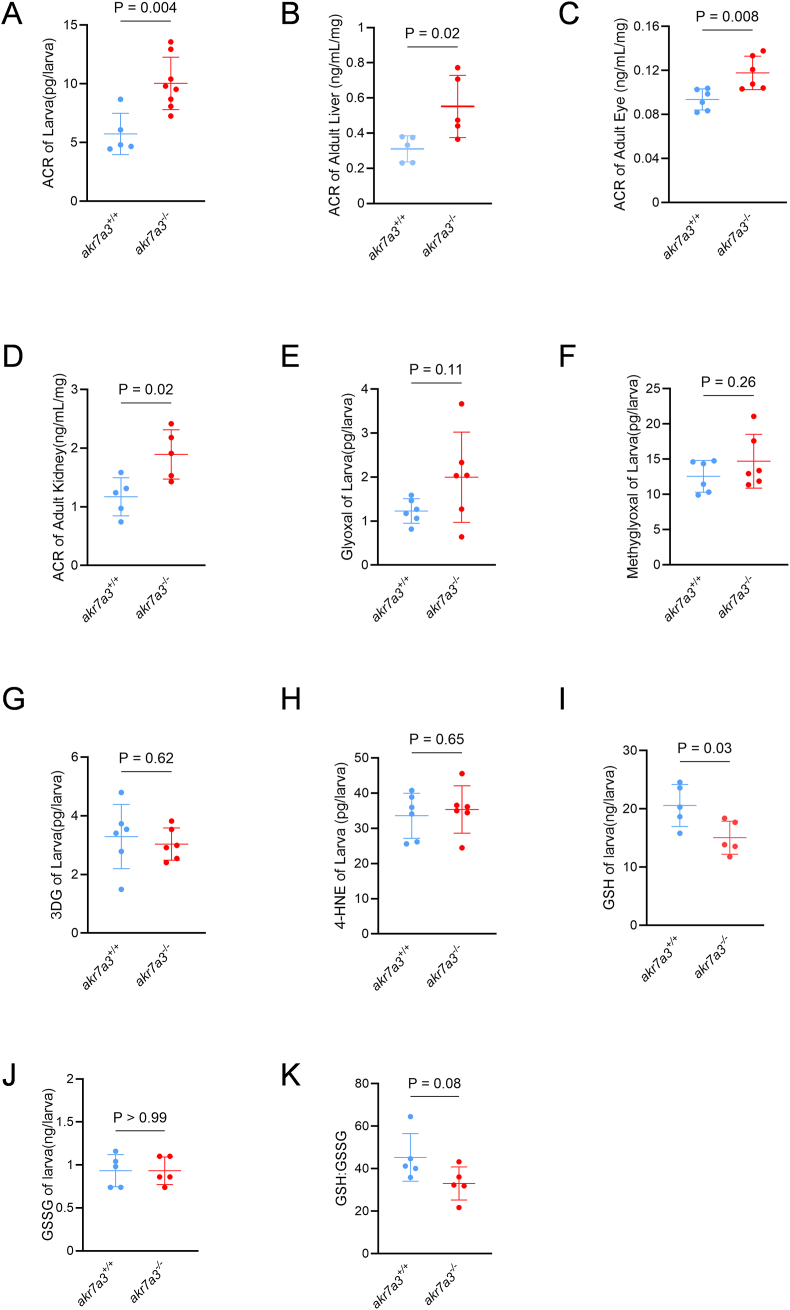
**Endogenous ACR was accumulated in *akr7a3*^*−/−*^ mutant zebrafish.** (A–D) ELISA determination indicated that ACR was significantly increased in 96 hpf old *akr7a3*^*−/−*^ larvae (A) and in adult *akr7a3*^*−/−*^ liver (B), *akr7a3*^*−/−*^ eye (C), and *akr7a3*^*−/−*^ kidney (D). Each data point indicates 47–50 larvae per clutch or one adult zebrafish organ, n = 5–8. (E–H) Representative RCS quantification by mass spectrometry or ELISA. The concentration of glyoxal (E), methylglyoxal (F), 3DG (G), and 4-HNE (H) remained unaltered in *akr7a3*^*−/−*^ larvae. Each data point indicates 47–50 larvae per clutch, n = 6. (I–K) Quantification of GSH and GSSG revealed a significant decrease in GSH in 5 dpf old *akr7a3*^*−/−*^ larvae (I), while GSSG (J) levels and the GSH/GSSG ratio (K) remained unchanged. Data represent 50 larvae per clutch, n = 5. The bars indicate mean ± SD values. Statistical analysis was performed by Student's t-test. ACR, acrolein; 3DG, 3-Deoxyglucosone; 4-Hydroxynonenal, 4-HNE; GSH, glutathione; GSSG, glutathione disulfide.

**Fig. 2:**
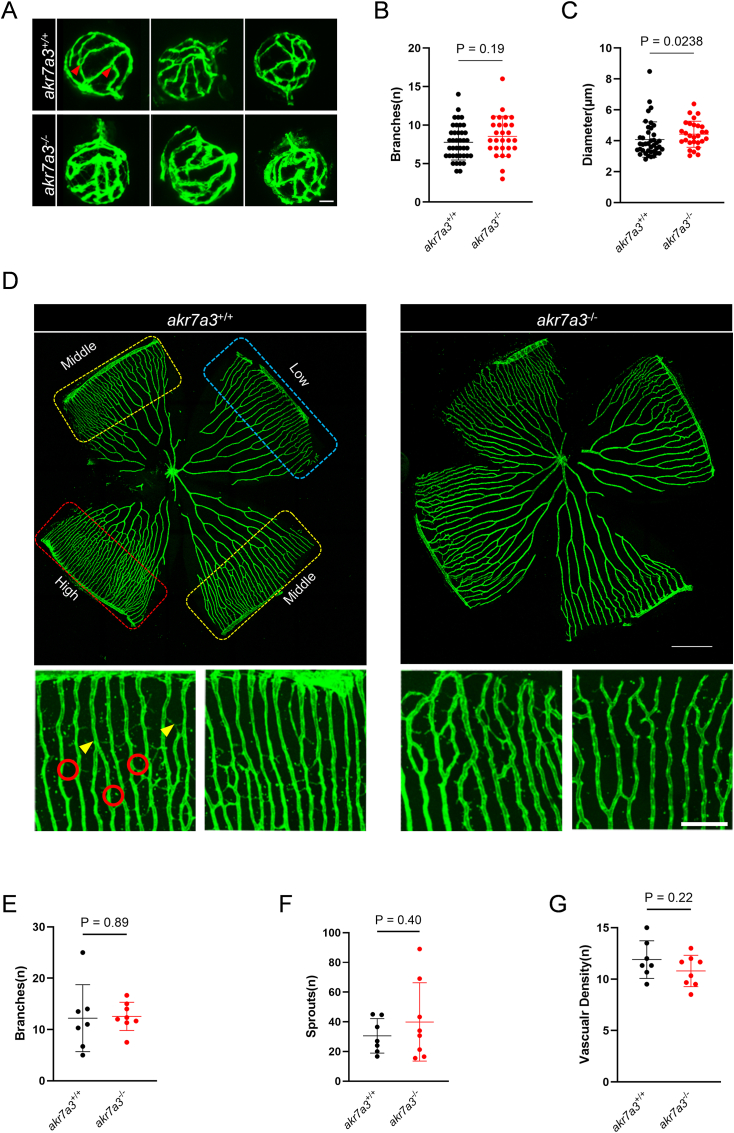
**Increased hyaloid vessel diameter in larvae of *akr7a3*^*−/−*^ eyes**. (A–C) Representative confocal images and quantification of hyaloid vasculature in larvae at 5dpf. No significant difference was observed in branches among *akr7a3*^*+/+*^ and *akr7a3*^*−/−*^ mutant larvae (B), however the diameter of hyaloid blood vessels was significantly increased in *akr7a3*^*−/−*^ mutants (C), white scale bar = 30 μm, n = 40/28, red arrows indicate branch points. (D–G) Representative confocal images and quantification of adult retinal vasculature. The vascular branches (E), sprouts (F), and vascular density (G) were not altered between adult *akr7a3*^*+/+*^ and *akr7a3*^*−/−*^ zebrafish. Yellow arrows are branch points, red circles are sprouts. Red frame, high density area; Blue frame, low density area; Yellow frame, middle density area; Upper white scale bar = 350 μm, lower white scale bar = 100 μm, n = 7/8. Each data point represents vasculature in a 350 μm^2^ area in the high-density region of the retina. Statistical analysis was performed by Student's t-test, The bars indicate mean ± SD values; dpf, days post fertilization.

**Fig. 3:**
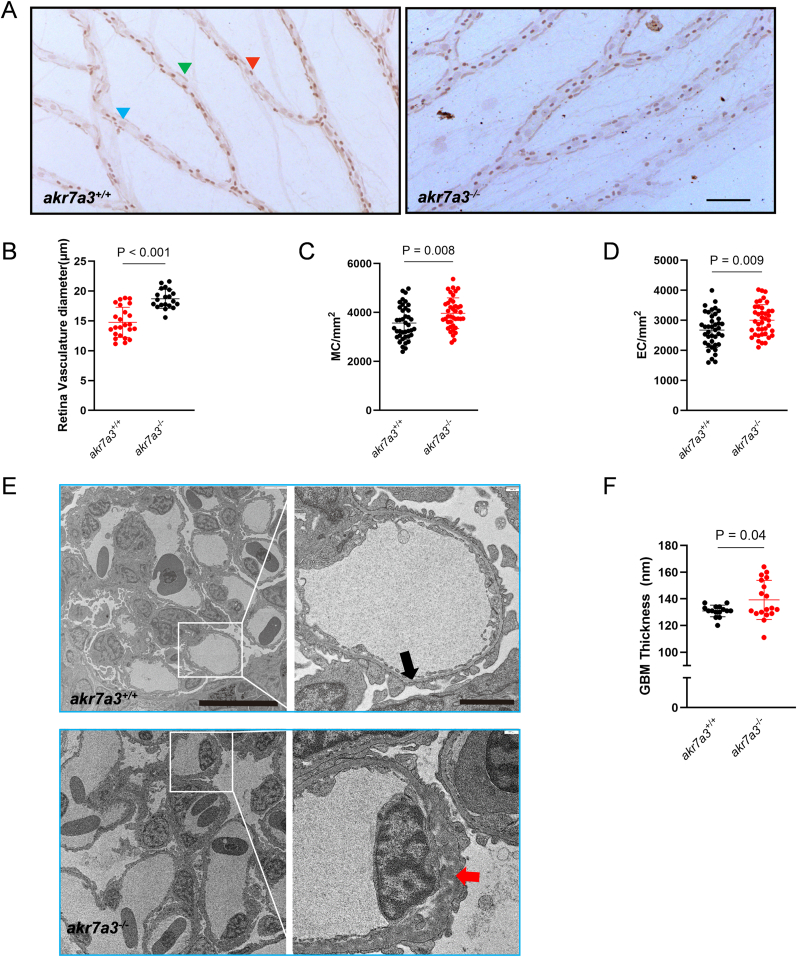
**Increased retinal vessel diameters and GBM thickening in adult *akr7a3*^*−/−*^ zebrafish.** (A–D) Representative hematoxylin staining and quantification of retinal vasculature. The retinal blood vessel diameters of *akr7a3*^*−/−*^ eyes were significantly increased (B). The number of MC (C) and EC (D) was also increased. n = 40. Blue arrows, EC; green arrows, MC; Red arrows, RBC; Black scale bar = 100 μm. (E,F) Representative EM images and GBM quantification showed a significant increase in GBM thickness in adult *akr7a3*^*−/−*^ kidneys. n = 15/18. Black arrow, normal GBM of adult *akr7a3*^+/+^ zebrafish, red arrow, thickened GBM of *akr7a3*^*−/−*^ zebrafish. Scale bar = 10 μm (left) and 2 μm (right), respectively. The bars indicate mean ± SD values. Statistical analysis was performed by Student's t-test. EC, endothelial cells; MC, mural cell; RBC, red blood cell; EM, electron microscopy; GBM, glomerular basement membrane.

To determine whether RCS were altered in *akr7a3*^*−/−*^ zebrafish, measurements of reactive metabolites including Acrolein (ACR), 4-Hydroxynonenal (4-HNE), Methylglyoxal (MG), Glyoxal, 3-Deoxyglucosone (3DG) were performed in zebrafish larvae and adults. The results showed that only ACR was significantly elevated in *akr7a3*^*−/−*^ zebrafish larvae to 10pg/larvae (Fig. 1A) compared to 6pg/larvae in *akr7a3*^*+/+*^ zebrafish. In addition, ACR was also increased in liver, eye, and kidney of adult *akr7a3*^*−/−*^ zebrafish (Fig. 1B–D). The other reactive metabolites measured, including glyoxal, MG, 3DG and 4-HNE (Fig. 1E–H), were not changed. Interestingly, glutathione (GSH) levels were decreased in *akr7a3*^*−/−*^ mutants while glutathione disulfide (GSSG) and the GSH:GSSG ratio remained unchanged (Fig. 1I–K). The reduction in GSH suggests a decreased redox buffering capacity in the *akr7a3* mutant, but since GSSG levels remain unchanged, the overall shift appears to be due to decreased GSH rather than increased oxidation per se. Taken together, these results identified ACR as the primary reactive metabolite that accumulates in *akr7a3*^*−/−*^ zebrafish. This accumulation persists throughout zebrafish development and is particularly evident in organs damaged in *akr7a3*^*−/−*^ zebrafish (see Fig. 2, Fig. 3).

### 2.3 Accumulated ACR altered vasculature in the eyes and glomerular basement membrane in *akr7a3*^−/−^ zebrafish

To determine whether the microvasculature was altered in *akr7a3*^*−/−*^ zebrafish, the vasculature of the eyes and the glomerular basement membrane (GBM) of the kidney were analyzed. Confocal microscopy data of the hyaloid vasculature showed no alteration in the number of vessel branches, but the diameters were increased (Fig. 2A–C). The adult retinal vasculature, including vessel sprouting, vessel branching and vessel density, was not significantly changed in adult *akr7a3*^*−/−*^ zebrafish (Fig. 2D–G). However, consistent with the increased vessel diameter of hyaloids in *akr7a3*^*−/−*^ larvae, the hematoxylin staining analysis confirmed an increased diameter of retinal blood vessels in adult *akr7a3*^*−/−*^ zebrafish (Fig. 3A and B). In addition, the number of mural cells (MC) and endothelial cells (EC) increased by 12.3 % and 11.1 %, respectively compared with *akr7a3*^*+/+*^ zebrafish (Fig. 3C and D). The GBM, located between the podocytes and the ECs, is a critical part of the filtration system, and GBM thickening is an early hallmark of metabolically driven renal changes, including diabetic nephropathy [30]. Transmission electron microscopy (TEM) analysis was performed to measure the thickness of the GBM and showed a significant increase in GBM thickness in adult *akr7a3*^*−/−*^ zebrafish kidneys (Fig. 3E and F). These data suggested that vascular structures are altered in the presence of endogenous ACR.

**Fig. 4:**
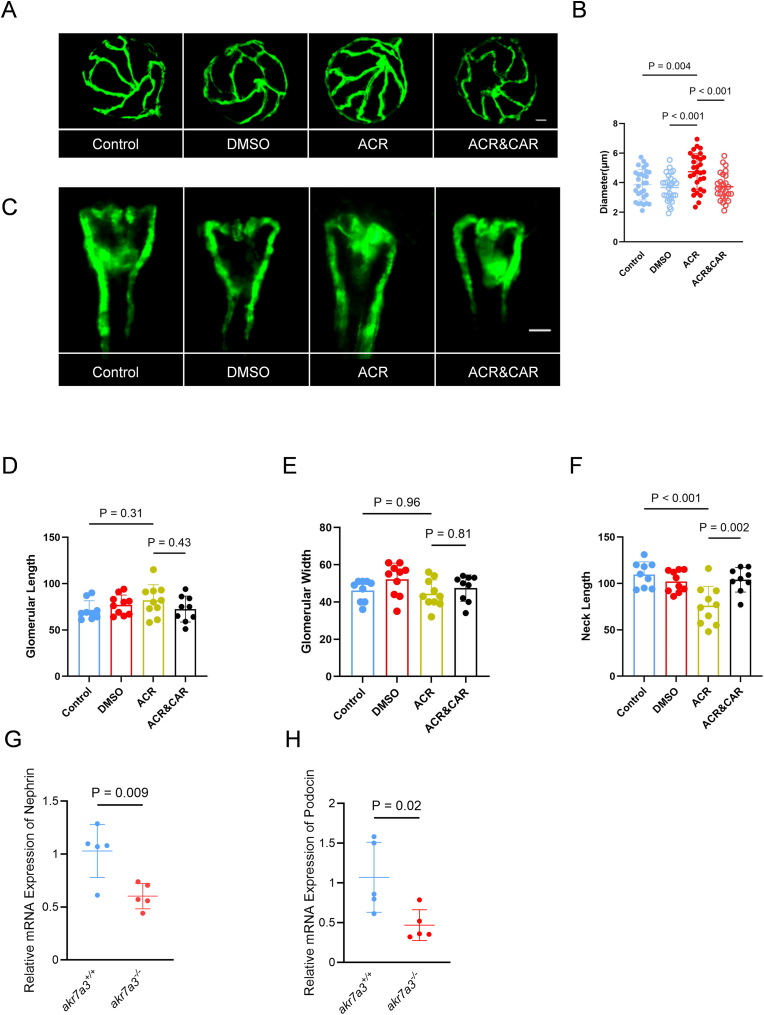
**Exogenous ACR induced dilation of hyaloid blood vessels and pronephric nephron alterations in zebrafish.** (A–B) Representative confocal images and quantification of hyaloid vasculature in larvae at 5dpf. A significant increase in diameter of hyaloid vasculature was observed in *akr7a3*^*+/+*^ larvae incubated with 10 μM ACR. n = 27–30, white scale bar = 30 μm. (C) Fluorescence microscopy images of pronephric nephrons at 48hpf. Larvae of *akr7a3*^*+/+*^ were incubated with ACR, ACR&CAR, or equivalent volume of DMSO as indicated, white scale bar = 50 μm. (D–F) Quantification of pronephric nephron indicated unchanged glomerular length (D) and glomerular width (E). However, neck length (F) was significantly reduced after ACR incubation compared to control group, while the reduced neck length was rescued in ACR&CAR co-incubation group. n = 9/10. (G–H) Expression levels of nephrin (G) and podocin (H) were significantly reduced in adult *akr7a3*^−/−^ kidneys, indicating a marked disruption of the glomerular filtration barrier in the absence of Akr7a3. n = 5. For statistical analysis, one-way ANOVA was used for comparisons among multiple groups, while the Student's t-test was applied for comparisons between two groups. Data are presented as mean ± SD. CAR, l-Carnosine; DMSO, Dimethyl sulfoxide.

To establish a causal relationship between the increased ACR levels in *akr7a3* mutants and the observed vascular changes, zebrafish embryos and larvae were incubated with ACR (Fig. S6) and the hyaloid vasculature and pronephros were analyzed. The results showed a significant dilation of the hyaloid vasculature after ACR treatment compared to controls. To demonstrate that acrolein itself mediates this effect, co-incubation with the ACR scavenger l-carnosine (CAR) was performed and it was found that CAR reversed the observed phenotype to a level compared to control larvae (Fig. 4A and B). In the pronephros, ACR decreased neck length, but co-incubation with CAR normalized it (Fig. 4C–F). Analysis of podocin and nephrin expression by qPCR in adult *akr7a3*^*−/−*^ kidneys as markers of renal physiology and function revealed reduced expression of both markers, indicating deterioration of renal function in the *akr7a3* mutant (Fig. 4G and H). Taken together, these data provide strong evidence that the accumulation of ACR is responsible for the phenotypes observed in the *akr7a3*^*−/−*^ mutant.

### 2.4 Increased arachidonic acid metabolism caused vascular changes in *akr7a3*^*−/−*^ zebrafish

**Fig. 5:**
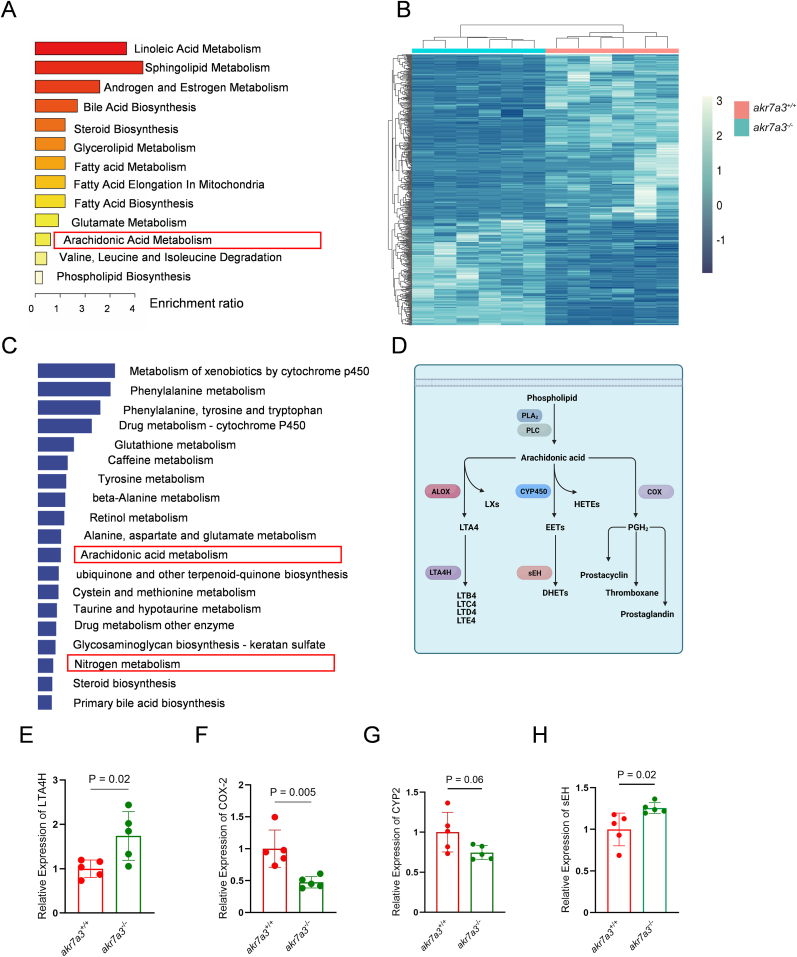
**Altered arachidonic acid metabolism pathway in *akr7a3*^−/−^ zebrafish.** (A) Pathway enrichment analysis based on metabolomic data indicates loss of *akr7a3* altered multiple metabolism pathways, most of which are involved in lipid metabolism. (B) Clustering analysis of RNA-seq disclosed that the gene expression profiles were significantly altered in adult eyes between *akr7a3***^−/−^** and *akr7a3***^+/+^** zebrafish. (C). GO/KEGG enrichment analysis of RNA-seq confirmed the alteration of lipid metabolic pathway, however the RNA-seq data also disclosed considerable metabolic changes of amino acids, glucose, and antioxidants between *akr7a3***^−/−^** and *akr7a3***^+/+^** zebrafish eyes as indicated. (D) Brief illustration of arachidonic acid metabolism pathway. Arachidonic acid is released from membrane phospholipids by the action of enzymes like PLA_2_/PLC and can then undergo various metabolic pathways and produces several bioactive lipid mediators include PGs, EETs, DHETs, and LTs. (E–H) Based on pathway enrichment analysis, expression of downstream targets gene including LTA4H, sEH, COX-2, and CYP2 was analyzed. Quantification of mRNA expression indicated an increase of LTA4H and sEH (E,H), a decrease of COX-2 (F), and an unchanged CYP2 in *akr7a3***^−/−^** mutants (G). n = 5 clutches with 30 larvae and n = 5 the adult liver and eye group. Student's t-test was applied for statistical analysis. PLA_2_/PLC, Phospholipase A2/C; LTA4H, Leukotriene A4 hydrolase; sEH, soluble epoxide hydrolase; COX-2, Cyclooxygenase-2; CYP2, Cytochrome P450 2. PGs, Prostaglandins; EETs, Epoxyeicosatrienoic acids; DHETs, Dihydroxyeicosatrienoic acids; LTs, Leukotrienes; HETEs, Hydroxyeicosatetraenoic acids; PGH_2_ Prostaglandin H2; ALOX, Arachidonate 5-lipoxygenase; LXs, Lipoxins; LTB4/C4/D4/E4, Leukotriene B4/C4/D4/E4.

To elucidate the relationship between ACR accumulation and vasodilation in *akr7a3*^*−/−*^ zebrafish, *akr7a3*^+/+^ and *akr7a3*^*−/−*^ zebrafish larvae, adult livers and eyes were subjected to metabolomic and transcriptomic analysis, as the liver showed the highest Akr7a3 expression and the eyes a strong vascular phenotype. In the metabolomic analysis, 630 target metabolites were analyzed; 469 metabolites were above the limit of detection (LOD); 146 metabolites were significantly altered between *akr7a3*^*+/+*^ and *akr7a3*^−/−^ zebrafish larvae samples (Fig. S3A), and hierarchical clustering analysis revealed that metabolites were clustered according to molecular structure and biological function (Fig. S3B). Metabolomic pathway enrichment analysis further showed that pathways such as steroid biosynthesis, fatty acid metabolism, and arachidonic acid metabolism as a precursor of leukotriene production were increased, which may subsequently be involved in the vascular alteration observed in the *akr7a3*^*−/−*^ zebrafish mutant (Fig. 5A).

As metabolomic changes are at least partially caused by altered gene expression, we also analyzed the transcriptome of *akr7a3* zebrafish larvae, liver and eyes (Fig. 5B–S4A) and performed KEGG/GO pathway enrichment analysis based on the transcriptome data. Increased arginine biosynthesis and nitrogen metabolism were identified as candidate pathways that may induce vascular alterations in *akr7a3*^−/−^ zebrafish (Fig. 5C–S4C). To test the hypothesis that nitric oxide (NO) accumulation is the primary factor driving vasodilation in *akr7a3*^−/−^ zebrafish, we utilized *N*_ω_-Nitro-l-Arginine Methyl Ester hydrochloride (l-NAME), an inhibitor of nitric oxide production, to block the effects of the arginine biosynthesis. However, analysis of the hyaloid vasculature showed that vessel diameter was not reduced in *akr7a3*^−/−^ zebrafish larvae incubated with either 5 μM or 20 μM l-NAME (Figs. S5C–F). This suggested that the arginine biosynthesis or nitrogen metabolism pathways were not responsible for the previous observations in *akr7a3*^−/−^ zebrafish, prompting us to explore alternative pathways.

**Fig. 6:**
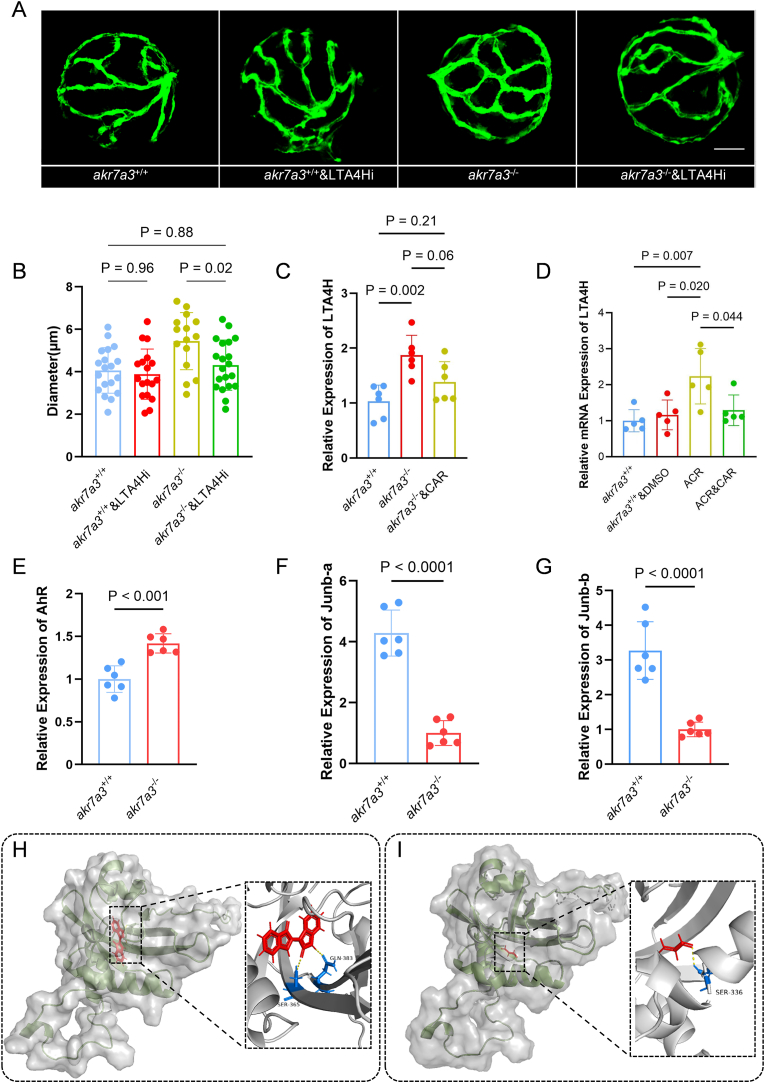
**LTA4H was upregulated by ACR and induced vascular alteration in *akr7a*3^−/−^ zebrafish.** (A–B) Representative confocal images and quantification of hyaloid blood vessel diameters in larvae at 5dpf. The increased diameter of hyaloid vasculature in *akr7a3*^−/−^ zebrafish was significantly reduced after LTA4Hi incubation. White scale bar = 30 μm, n = 18–20. (C–D) RT-qPCR analysis of LTA4H expression. LTA4H was significantly increased in *akr7a3*^−/−^ zebrafish larvae. Incubation with CAR reduced the expression of LTA4H non-significantly. After incubating *akr7a3*^*+*/+^ zebrafish larvae with ACR, an increase in LTA4H expression was observed. However, co-incubation with ACR and CAR was able to inhibit the elevated expression of LTA4H. n = 6 clutches with 30 larvae. (E–G) RT-qPCR analysis data revealed an increased expression of AhR (E), while Junb-a (F) and Junb-b (G) exhibited decreased expression. n = 6 clutches with 30 larvae. (H–I) Docking analysis showed ACR (I) shared same binding pocket with Indirubin (H). ACR formed a hydrogen bond with the amino residue SER336 of AhR. Dash line = hydrogen bonds. For statistical analysis one-way ANOVA or Student's t-test was applied. The bars indicate values of mean ± SD. AhR, Aryl hydrocarbon receptor; Junb-a, JunB Proto-Oncogene a; Junb-b, JunB Proto-Oncogene b.

The arachidonic acid metabolism pathway was also markedly enriched in both transcriptome and metabolome analyses (Fig. 5A–C,D and S4B). Arachidonic acid is used as a substrate by cyclooxygenases (COXs), lipoxygenases (LOXs), and cytochrome P450 (CYP) enzymes leading to the generation of a variety of different bioactive mediators [31]. This spectrum includes prostanoids, leukotrienes (LTs), epoxyeicosatrienoic acids (EETs), and dihydroxyeicosatrienoic acids (DHETs), suggesting a potential function for arachidonic acid metabolism in the vascular alteration observed in *akr7a3*^−/−^ zebrafish [32]. While cyclooxygenase-2 (COX-2) mRNA expression was reduced in *akr7a3*^−/−^ zebrafish, cytochrome P450 2 (CYP2) expression remained unchanged (Fig. 5F and G). However, the expression of leukotriene A4 hydrolase (LTA4H) and soluble epoxide hydrolase (sEH) was increased in *akr7a3* mutants (Fig. 5E–H), suggesting that upregulation of the LTs and EETs pathway may be the potential mechanism for vascular alterations in *akr7a3*^−/−^ zebrafish. Therefore, hyaloid vasculature was analyzed using pharmacological inhibitors of both pathways. The diameters of the hyaloid vasculature remained unchanged between *akr7a3*^−/−^ and *akr7a3*^−/−^ zebrafish larvae incubated with 20 μM epoxide hydrolase inhibitor (sEHi) (Figs. S5G and H), suggesting that upregulation of sEH was not responsible for the vasodilation of the hyaloid vasculature in *akr7a3*^−/−^ zebrafish larvae. In contrast, the leukotriene A4 hydrolase inhibitor (LTA4Hi) reduced the hyaloid vessel diameter in *akr7a3*^−/−^ zebrafish back to normal values (Fig. 6A,B and S5A,B).

To establish a causal link between ACR and LTA4H on vascular function, its rescue by CAR treatment and its transcriptional regulation, we analyzed the expression of LTA4H in *akr7a3*^−/−^ zebrafish larvae incubated with CAR and found decreased LTA4H levels, although not statistically significant (Fig. 6C). Furthermore, ACR increased LTA4H expression in *akr7a3*^+/+^ zebrafish larvae, which was inhibited after co-incubation with CAR (Fig. 6D). The transcription factors aryl hydrocarbon receptor (AhR), which is known to be involved in lipid mediator production [33,34], was increased in *akr7a3*^−/−^ zebrafish eyes (Fig. 6E), whereas the expression of Junb-a and Junb-b was decreased (Fig. 6F and G). These results suggested that the activation of AhR may be responsible for the upregulation of LTA4H. To clarify a possible interaction between ACR and AhR, a molecule-protein docking analysis was performed. The docking analysis revealed that ACR shares a similar binding pocket with the AhR agonist indirubin and within this binding pocket (Fig. 6H), the aldehyde group of ACR forms a hydrogen bond with the amino residue SER336 of AhR (Fig. 6I), suggesting that ACR has a strong affinity and similar activity as indirubin, indicating that ACR acts as an agonist of AhR and can promote LTA4H expression as well as LTs production.

**Fig. 7:**
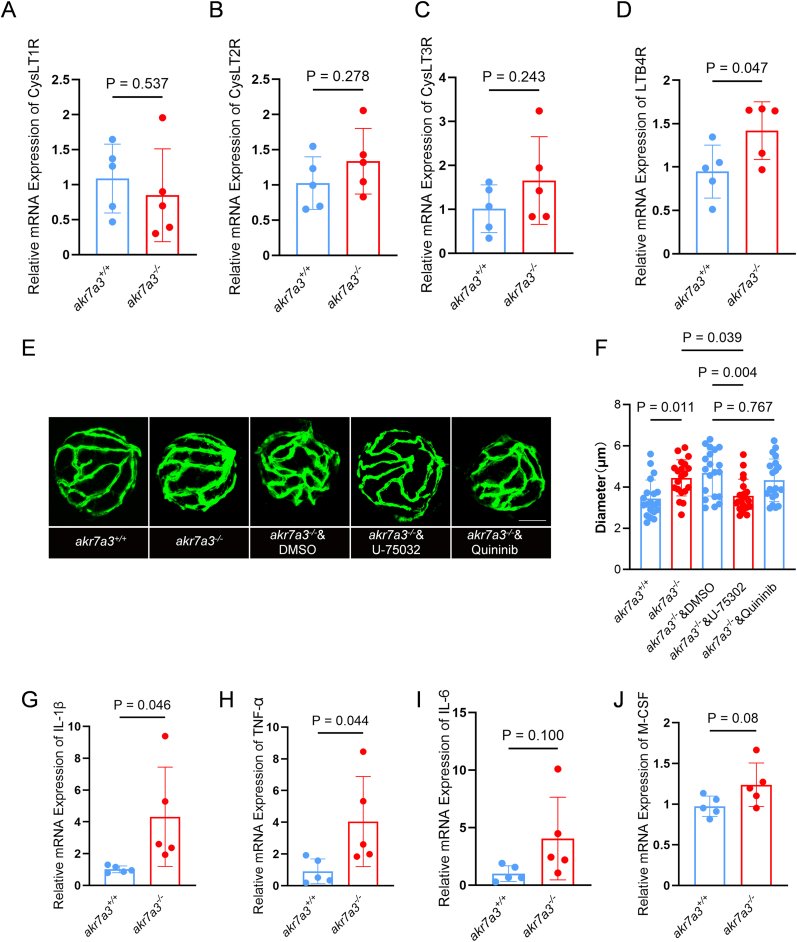
**Upregulated activity of LTB4R promoted inflammatory response and vascular alteration in *akr7a*3^−/−^ zebrafish.** (A–D) The expression of leukotriene receptors was examined. It revealed that the expression of CysLT1R (A), CysLT2R (B), and CysLT3R (C) were not altered but most gene showed an upregulated trend in *akr7a3*^−/−^ group whereas LTB4R (D) was significantly overexpressed. n = 5 clutches with 30 larvae. (E–F) Rescue experiment using antagonist for LTB4R and CysLTs: Antagonists of LTB4R (U-75032) and CysLTs (Quininib) were used to incubate *akr7a3*^−/−^ larvae. Treatment with U-75032 significantly reduced the retinal vessel diameter in *akr7a3*^−/−^ larvae, whereas Quininib had no significant effect on the retinal vessel diameter in these larvae. n = 20 larvae. (G–J) Representative inflammatory factors were also analyzed with RT-qPCR. IL-1β (G) and TNF-α (H) were significantly overexpressed while IL-6 (I) and M-CSF (J) were increased non-sigificantly in *akr7a3*^−/−^ group. n = 5 clutches with 30 larvae.The bars indicate mean ± SD values. Statistical analysis was performed by one-way ANOVA or Student's t-test. CysLTs, Cysteinyl leukotriene receptor 1 (CysLT1R), 2 (CysLT2R), and 3 (CysLT3R); LTB4R, Leukotriene B4 receptor; IL-1β, Interleukin-1 beta; TNF-α, Tumor Necrosis Factor-alpha; M-CSF, IL-6, interleukin 6; Macrophage Colony-Stimulating Factor.

Since LTs can induce vasodilation and inflammatory responses via several leukotriene receptors, RT-qPCR analysis was performed for several LTs receptors and key inflammatory cytokines. The data showed that leukotriene receptors, including cysteinyl leukotriene receptor 1 (CysLT1R), cysteinyl leukotriene receptor 2 (CysLT2R), and cysteinyl leukotriene receptor 3 (CysLT3R) did not show significant changes (Fig. 7A–C) while Leukotriene B4 receptor (LTB4R) was upregulated in *akr7a3*^−/−^ zebrafish eyes (Fig. 7D). To elucidate the biological effects of enhanced LTB4R expression, we performed an interventional hyaloid vessel analysis together with an examination of several key inflammatory factors, including Interleukin-1 beta (IL-1β), tumor necrosis factor-alpha (TNF-α), and macrophage colony-stimulating factor (M-CSF). Antagonists of LTB4R (U-75032) and CysLTs (Quininib) were used to assess the potential rescue effects of blocking LTs receptors in *akr7a3*^−/−^ larvae. Treatment with U-75032 significantly reduced the diameter of the hyaloid vasculature in *akr7a3*^−/−^ larvae, whereas Quininib had no significant effect (Fig. 7E and F). Finally, examination of several key inflammatory cytokines showed that IL-1β and TNF-α were increased in *akr7a3*^−/−^ zebrafish eyes (Fig. 7G and H) whereas interleukin-6 (IL-6) and M-CSF showed an increasing trend but without statistical significance (Fig. 7I and J).

Altogether, the above data have identified a novel cascade starting with endogenously accumulated ACR in *akr7a3*^*−/−*^ mutants, which subsequently activates an AhR-mediated LTA4H expression, leading to inflammation and microvascular dysfunction.

## 3 Discussion

In this study, an *akr7a3*^*−/−*^ knockout zebrafish line was established, ACR was identified as a substrate, and endogenously accumulated ACR damaged ocular vascular integrity and renal structure. Mechanistically, elevated ACR does not primarily activate endothelial cell dysfunction via a general non-specific post-translational modification, but rather via increased arachidonic acid metabolism and LTA4H expression. Thus, a novel AKR-ACR-AhR-LTs triggered pathway of endothelial cell dysfunction has been discovered.

ACR is known for its detrimental role in inducing oxidative stress, initiating inflammation and damaging cellular components, but the lack of specific acrolein inhibitors has made it difficult to investigate its causal role and key targets in disease-related organ changes. In this study, AKR7a3 was identified as an ACR detoxification system because ACR was endogenously elevated in the zebrafish retinal vasculature, resulting in an increase in vessel diameter. This change was found in both larval and adult zebrafish, suggesting that the vasodilatation is a long-term structural change along with the continuous accumulation of ACR. However, the vasodilatory effect of ACR was not mediated by increased NO production, as recently shown in mouse mesenteric artery [35], since the use of l-NAME, an inhibitor of NO formation, failed to block vasodilation in the hyaloid vasculature of *akr7a3*^−/−^ zebrafish larvae. Instead, transcriptomic and metabolomic data revealed that ACR led to an up-regulation of the arachidonic acid pathway, which serves as a precursor for the production of leukotrienes, some of which have vasodilatory activity. Leukotriene A4 hydrolase was upregulated in the *akr7a3* mutant zebrafish and by direct treatment with ACR, and blocking the function of the LTB4 receptors reversed the vascular changes suggesting increased leukotriene formation and an inflammatory response as the cause of the vasodilatory phenotype in the vasculature. Molecular docking analysis suggested that AhR may mediate ACR-induced overexpression of leukotriene A4 hydrolase. Consistent with our findings, previous studies have reported that ACR can directly activate AhR [36]. Further research has also shown that AhR activation promotes the production of arachidonic acid metabolites, including LTB4 [34,37]. In addition to changes in the ocular vasculature, our results also showed thickening of the GBM in the kidney of *akr7a3*^−/−^ zebrafish, and both changes could be recapitulated by the addition of external ACR to wild-type zebrafish. Thus, our study identified ACR as a primary damaging factor to the eye and kidney in zebrafish.

The ACR-induced significant increase in the number of endothelial cells (EC) and mural cells (MC) was found together with vasodilation in the adult retinal vasculature. This finding is consistent with other studies that have reported that ACR is involved in fibrovascular proliferation in patients with proliferative diabetic retinopathy (PDR) [38,39]. In addition, incubation of human retinal microvascular endothelial cells with a sublethal dose of ACR resulted in increased cell proliferation [38]. ACR can induce transforming growth factor beta-1 (TGF-β1), TGF-β2 and vascular endothelial growth factor (VEGF) production under hyperglycaemic conditions [40]. These findings support our observation of ACR-induced retinal vascular changes in *akr7a3*^−/−^ zebrafish. An increased number of MCs, consisting of smooth muscle cells (SMCs) and pericytes, is an additional effect of ACR in the zebrafish retina, while previous studies reported that ACR could activate inflammatory signalling in vascular SMCs [41] and induce a relaxation response in mouse tracheal smooth muscle in a dose-dependent manner [42]. In addition to our observations, other retinal abnormalities associated with ACR accumulation have also been reported. Suneel et al. [43] reported that ACR exposure disrupted all three corneal layers—the epithelium, stroma, and endothelium—in a rabbit model. Acrolein exposure resulted in severe ocular surface abrasions in the epithelial cell layer and significant alterations in the stromal layer, including edema and infiltration by inflammatory cells. Moreover, conjugated ACR was associated with the retinal thinning, neurodegeneration, and abnormal visual function in diabetic animals [44,45]. Murata and colleagues reported that ACR promoted retinal Müller glial cell migration by enhancing C-X-C motif chemokine ligand 1 (CXCL1) production, which eventually could contribute to severe visual dysfunction in patients with PDR. Collectively, ACR can induce various abnormalities in retina via different mechanisms. Our observations in *akr7a3*^*−/−*^ zebrafish provide new insights into the role of ACR in regulating vascular integrity and further our understanding of its impact on retinal health.

Accumulation of endogenous ACR can also induce epithelial-to-mesenchymal transition (EMT) and abnormal glycolysis via TGF-β signalling in tubular cells of diabetic mice, contributing to renal fibrogenesis. This highlights the role of ACR and TGF-β signalling in promoting metabolic and cellular changes associated with renal fibrosis in diabetic conditions [5]. The ACR-induced GBM thickening in the *akr7a3*^*−/−*^ mutant further supports the damaging capacity of ACR on the kidney. Other studies have reported that ACR increases inflammatory cytokines and cellular apoptosis in human renal cell lines [7], while hypoxia-reoxygenation-induced acrolein accumulation exacerbates acute kidney injury by promoting tubular cell death [8], which together suggest that ACR plays a detrimental role in disrupting renal metabolism and impairing renal function.

In conclusion, this study has demonstrated a causative role of ACR in the pathogenesis of vascular disease by promoting endothelial dysfunction through increased activation of the arachidonic acid pathway. Therefore, ACR could be considered as a potential therapeutic target for the treatment of vascular disease.

## 4 Material and methods

### 4.1 Study approval

All animal-related experiments and procedures has been approved by the Regierungspräsidium Karlsruhe (license no. G-98/15) and Heidelberg University (I-21/19 and I-24/17). These experiments were carried out in full compliance with the approved guidelines, including adherence to the EU Directive 2010/63/EU on the protection of animals used for scientific purposes.

### 4.2 Zebrafish husbandry

The *Tg(fli1:EGFP)* and *Tg(wt1b:EGFP)* zebrafish lines was raised and staged following established protocols [46,47]. Embryos/larvae were staged in hours post-fertilization (hpf) or days post-fertilization(dpf). 0.003 % PTU (N-Phenylthiourea, CAS 103-85-5) was added to the environment to suppress pigmentation for imaging studies. Embryos/larvae were kept in E3 medium at 28.5 °C Until 5 dpf. The adult zebrafish and Larvae older than 5dpf were maintained under a 13-h light and 11-h dark cycle and were fed twice daily with freshly hatched Artemia salina and flake food, respectively.

### 4.3 Mutant generation

The CRISPR/Cas9 technique was adopted to generate *akr7a3*^−/−^ fishline, following a previously described procedure. The CRISPR guide-RNA (gRNA) was designed utilizing ZiFit-targeter Version 3.3 to target a specific site in exon 1 of *akr7a3* gene. This gRNA was then inserted into a pT7-gRNA promoter expression vector (Addgene) (primers are listed in Supplementary Table 1). To obtain Cas9 mRNA, the pT3TS-nCas9n Vector (Addgene) was used for in vitro transcription [48]. The Cas9 mRNA synthesis was carried out using the mMESSAGE mMACHINE T3 Transcription Kit following the manufacturers' protocols, while the MEGAshortscript T7 Kit (Invitrogen) was used for gRNA synthesis. Mutants were generated by injecting 1 nL of a 0.1 M KCl solution containing gRNA (200 pg nL^−1^) and Cas9 mRNA (200 pg nL^−1^) into *Tg(fli1:EGFP)* embryos at the 1-cell stage. Subsequently, the injected adult mosaic zebrafish (F0) were analyzed for germline transmission using Sanger sequencing of PCR products. Mutated fish were then bred with *Tg(fli1:EGFP)* zebrafish to produce heterozygous mutants. Genotyping of the mutants was performed through Sanger sequencing and gel electrophoretic separation of PCR products (primers are listed in Supplementary Table 1).

### 4.4 Preparation of adult zebrafish

After euthanasia, the zebrafish were promptly transferred to an experimental platform submerged in ice-cold PBS. Organs were then carefully collected, weighed, and immediately snap-frozen in liquid nitrogen before being stored at −80 °C for subsequent analysis. Alternatively, organs were excised and transferred into 4 % PFA for histology analysis. For ultrastructural analysis of kidney glomerular, kidneys were excised and immediately transferred to 3 % glutaraldehyde in 0.1 M cacodylate buffer (pH 7.4) for further processing.

### 4.5 Analysis of kidney morphology

Electron Microscopy was used to analyze the structure of kidney glomerular. The preparation was conducted in compliance of protocol as previously described. The structure of zebrafish kidney glomerular were analyzed by Electron Microscopy (EM). The preparation procedures followed the methods described earlier [17]. Imaging and processing were performed in collaboration with the Institute of Pathology at Heidelberg University Hospital (IPH).

### 4.6 Western blot analysis

For Western blot analysis, 30 larvae or adult organs were harvested and lysed using NP40 lysis buffer, which contained 1 % NP40, 10 mmol/L EDTA, 50 mmol/L Tris-HCl (pH 7.4), 10 % glycerol, 150 mmol/L NaCl, and protease inhibitors. The lysate was homogenized and then incubated on ice for 30 min with shaking. The lysate to was centrifuged to remove debris, then the resulting supernatant (protein extract) was collected and protein concentration was measured. The supernatant was mixed with Laemmli sample buffer at a 5:1 ratio and then heated at 95 °C for 5 min to denature the proteins. Load 30 μg of protein per well into the SDS-PAGE gel and run electrophoresis at 100 V until the dye front reaches the bottom. Next, the proteins were transferred to a nitrocellulose membrane for antibody incubation. For incubation, primary and secondary antibodies were used at a 1:1000 dilution, including anti-β-actin (Santa Cruz Biotechnology, sc-47778), anti-Akr7a3 (Invitrogen, PA5-100382), and HRP-conjugated secondary antibodies: rabbit anti-mouse (DAKO, P0260) for β-actin, and goat anti-rabbit (DAKO, P0448) for Akr7a3. After incubation, the target protein was detected through enhanced chemiluminescence (PerkinElmer) on a Vilber Fusion Solo S imaging system.

### 4.7 Reverse-transcription quantitative polymerase chain reaction analysis (RT-qPCR)

Larvae or adult organs were collected and immediately snap-frozen in liquid nitrogen for RNA isolation. Total RNA was extracted using the RNeasy Mini Kit (Qiagen) following the manufacturer's recommended protocol. Maxima First Strand cDNA Synthesis Kit (Thermo Fisher Scientific) was used to transcribe cDNA with 500 μg template RNA Primers for qPCR were designed using NCBI Primer-BLAST and synthesized by Sigma-Aldrich (see Supplementary Table 1 for primer details). RT-qPCR was performed using the Power SYBR Green PCR Master Mix Kit (Thermo Fisher Scientific) in 96-well plates, and amplification was carried out on a QuantStudio 3 Real-Time PCR System (Thermo Fisher Scientific).

### 4.8 Reactive metabolites assay

Reactive metabolites, including MG, glyoxal, and 3-DG, were detected following the protocol as previously described [[49], [50], [51]].

### 4.9 Glutathione and glutathione disulfide measurements

Glutathione (GSH) and glutathione disulfide (GSSG) levels were measured as previously described [52].

### 4.10 Enzyme-linked immunosorbent assay (ELISA)

Adult zebrafish livers, kidneys, eyes, and larvae at 96 hpf were collected and snap-frozen for quantitative measurement of ACR and 4-HNE. Adult organs or 40–50 larvae per clutch were homogenized in 1 × PBS. ACR (MyBioSource Inc) and 4-HNE (abcam) were detected according to protocol provided by manufacturer.

### 4.11 Imaging and quantitative analysis of vascular changes in zebrafish larvae and adults

To analyze the hyaloid vasculature, zebrafish larvae at 96 hpf were anesthetized and fixed in 4 % PFA for 24 h. Next, a 0.25 % Trypsin/EDTA solution buffered with TRIS HCl (1.5 M, pH 7.8) was used to incubate the larvae for 80 min at room temperature. Afterwards, the larvae were washed three times for 10 min with PBS. Then the hyaloids were excised and subjected to visualization according to previously described protocol [53]. Adult retina vasculature was analyzed using the method outlined by Wiggenhauser et al. [54]. Prior to dissection, the zebrafish eye was fixed in 4 % PFA buffer. After fixation, the eye was transferred to a pre-prepared agarose plate in a cold PBS environment. The retina was then dissected and placed on a slide for mounting. The mounted slide was covered with a cover slip and subsequently imaged using confocal fluorescence microscopy (Leica DM6000 B) with Leica TCS SP5 DS scanner for further analysis and processing.

### 4.12 Retinal digestion and hemalum staining

Vascular diameter, as well as the number of endothelial cell and pericytes are analyzed by retinal digestion and hemalum staining. The digestion was conducted following an established protocol with minor modifications [55]. Briefly, the eyes of the adult zebrafish were prepared as previously described [54]. After dissection, the retina was transferred to a Petri dish filled with aqua bidest and was incubated at 37 °C overnight. The retina was then incubated in a 3 % porcine trypsin (Sigma-Aldrich) solution in Tris-HCl (0.2 M) at 37 °C for 90 min. After incubation, the retina was transferred onto a microscope slide, where retinal cells were gently removed from the vasculature by dropping aqua bidest. The retina was then left to air-dry, allowing it to attach to the slide. Nuclei staining was conducted to visualize different cell types after retina preparation was completed. The slides were briefly placed in aqua bidest and then transferred into a freshly prepared Mayer's hemalum solution (Millipore) for 7 min. The slides were then placed in lukewarm tap water for 2 min. Following this, they were briefly soaked in 70 % ethanol, followed by 80 % ethanol. The slides were then sequentially immersed in 96 %, 100 %, and finally xylene for 5 min each. Finally, the slides were covered with cover slips using DPX mounting medium (Thermo Fisher Scientific) for subsequent imaging.

### 4.13 Microscopy and analysis of pronephric alterations

*Tg(wt1b:EGFP)* embryos were used to analyze the pronephric structures. Briefly, 48 hpf embryos were anesthetized with 0.003 % tricaine, mounted dorsally in 1 % low-melting-point agarose (Promega), and placed on an objective slide. The slide was then subjected to fluorescent imaging with a Leica MZ10 F modular stereo microscope. The slide was then imaged using a Leica MZ10 F modular stereo microscope. For quantitative analysis, the glomerular length, width, and neck size of the pronephric structures were measured as described by Wiggenhauser et al. [56].

### 4.14 RNA sequencing analysis

Adult zebrafish livers, eyes and larvae at 5dpf were collected and were subjected to RNA purification using the RNeasy Kit as previously described. Library construction and sequencing were conducted with DNBseq (Beijing Genomic Institution, www.bgi.com, BGI). High-quality reads were mapped to the Zebrafish reference genome (GRCz11) using HISAT2 software (v2.1.1). FeatureCounts (v2.0.3) was used to quantify expression reads levels. Further analysis including normalization, differential expression analysis, enrichment analysis and heatmap analysis were performed with edgeR (v3.40.2), limma package (v3.54.2), GSVA package (v1.46.0), and ggplot 2 package (version 3.5.1) respectively. The data can be accessed on https://www.ncbi.nlm.nih.gov/geo/query/acc.cgi?acc=GSE288313.

### 4.15 Metabolomic analysis

Zebrafish larvae at 96 hpf were collected for metabolomic detection. Each sample consists of 50 larvae, which were anesthetized with a 0.003 % tricaine solution and subsequently snap-frozen in liquid nitrogen. The detection was conducted in collaboration with e Metabolomics Core Technology Platform (MCTP) from the Centre of Organismal Studies Heidelberg. The detection of organic compounds includes adenosine compounds, thiols, free amino acids, fatty acids, and primary metabolites, with the methods as previously described [49].

### 4.16 Pharmacological treatment of zebrafish embryos/larvae

25-30 zebrafish embryos were placed into 6-well plates, with each well containing 5 mL of egg water. The chorions of the embryos were carefully removed at 24 hpf. For intervention and rescue experiments, the following compounds were used: 0–15 μM ACR (S–11030F1; CHEM SERVICE), 10 mM l-Carnosine (C9625; Sigma-Aldrich), 0–175 μM LTA4Hi for toxicity test, 30 μM LTA4Hi for incubation (HY-103226; MedChemExpress), 5 μM or 20 μM *N*_ω_-Nitro-l-Arginine Methyl Ester hydrochloride (N5751; Sigma-Aldrich), 20 μM sEHi (HY-113974; MedChemExpress), 500 nM U-75302 (119477-85-9; Cayman); 500 nM Quininib (143816-42-6; Cayman). The egg water was renewed each day to maintain a consistent level.

### 4.17 Molecular docking

The molecular structure of ACR was sourced from the PubChem Compound database (PubChem CID: 7847), while crystal structure of AhR were obtained from the Protein Data Bank (PDB ID: 7ZUB). The Benzo[*a*]pyrene-AhR complex (PDB ID: 8QMO) served as a positive control model for the comparative analysis. These models were examined following the methodology outlined in the published work [1].

### 4.18 Statistical analysis

Data are presented as the mean ± standard deviation. Unpaired Student's t-test or one-way ANOVA was used for comparisons among multiple groups. Statistical analysis was performed using GraphPad Prism 8.

## CRediT authorship contribution statement

**Xin Zhang:** Writing – original draft, Validation, Investigation, Formal analysis, Data curation. **Johannes Gschwind:** Investigation, Formal analysis, Data curation. **Vanessa Erben:** Investigation, Formal analysis, Data curation. **Katrin Bennewitz:** Methodology, Data curation. **Xiaogang Li:** Methodology, Investigation. **Carsten Sticht:** Formal analysis. **Gernot Poschet:** Investigation, Formal analysis. **Ingrid Hausser:** Investigation, Formal analysis, Data curation. **Thomas Fleming:** Investigation, Formal analysis, Data curation. **Julia Szendroedi:** Conceptualization. **Peter Paul Nawroth:** Conceptualization. **Jens Kroll:** Writing – review & editing, Supervision, Project administration, Funding acquisition, Conceptualization.

## Funding

The study was supported by grants from 10.13039/501100001659Deutsche Forschungsgemeinschaft (CRC1118 and project 517361638) and the 10.13039/501100004543China Scholarship Council (CSC 202208320073).

## Declaration of competing interest

The authors declare that they have no known competing financial interests or personal relationships that could have appeared to influence the work reported in this paper.

## Data Availability

Data will be made available on request.
