# Supplementary material for: Endogenous acrolein accumulation in akr7a3 mutants causes microvascular dysfunction due to increased arachidonic acid metabolism
Source: Redox Biol. 2025 Apr 17;83:103639. doi: 10.1016/j.redox.2025.103639 (PMC12051060; doi:10.1016/j.redox.2025.103639)
Supplement: Multimedia component 1 [file mmc1.pdf]

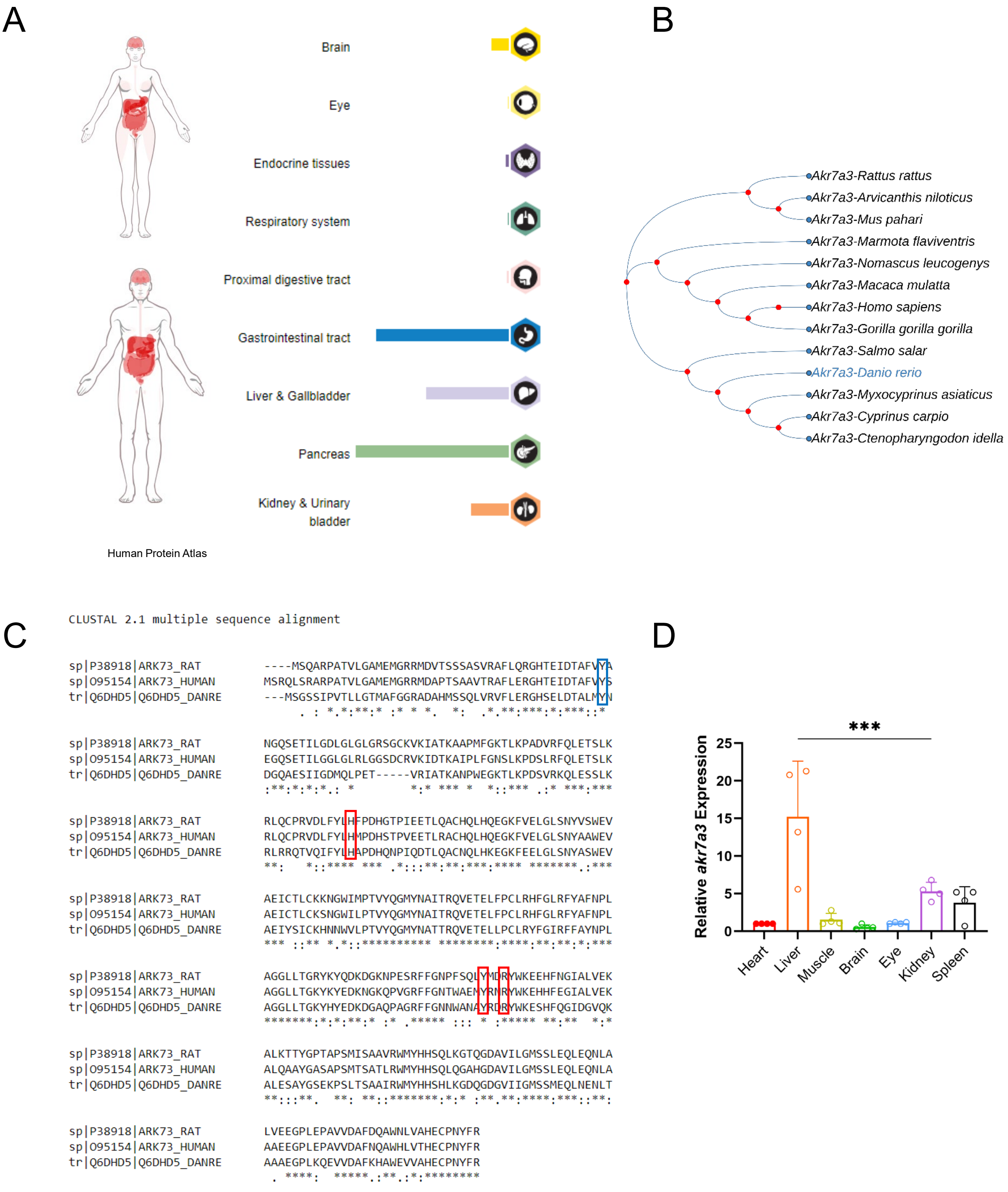

Figure S1. Expression and evolution of the *akr7a3* in human and zebrafish (A) Illustration depicting the tissue expression of *akr7a3* mRNA in the human body. (B) Neighbor-joining phylogenetic tree of amino acid sequences illustrates the evolutionary relationships of *akr7a3* among representative vertebrate species. (C) Amino acid alignment analysis showed zebrafish, human and rat shared same active site (blue frame) and substrates binding site (red frame) in Akr7a3 amino acid sequence. (D) RT-qPCR analysis indicated *akr7a3* was predominantly expressed in the liver, with minimal expression observed in the kidney and spleen in adult *akr7a3*$^{+/+}$ zebrafish (n= 4-6). Each data point in this figure represented one adult fish. The bars indicate values of mean ± SD. Statistical analysis was performed by one-way ANOVA or Student's t-test. arnt2, aryl hydrocarbon receptor nuclear translocator 2.

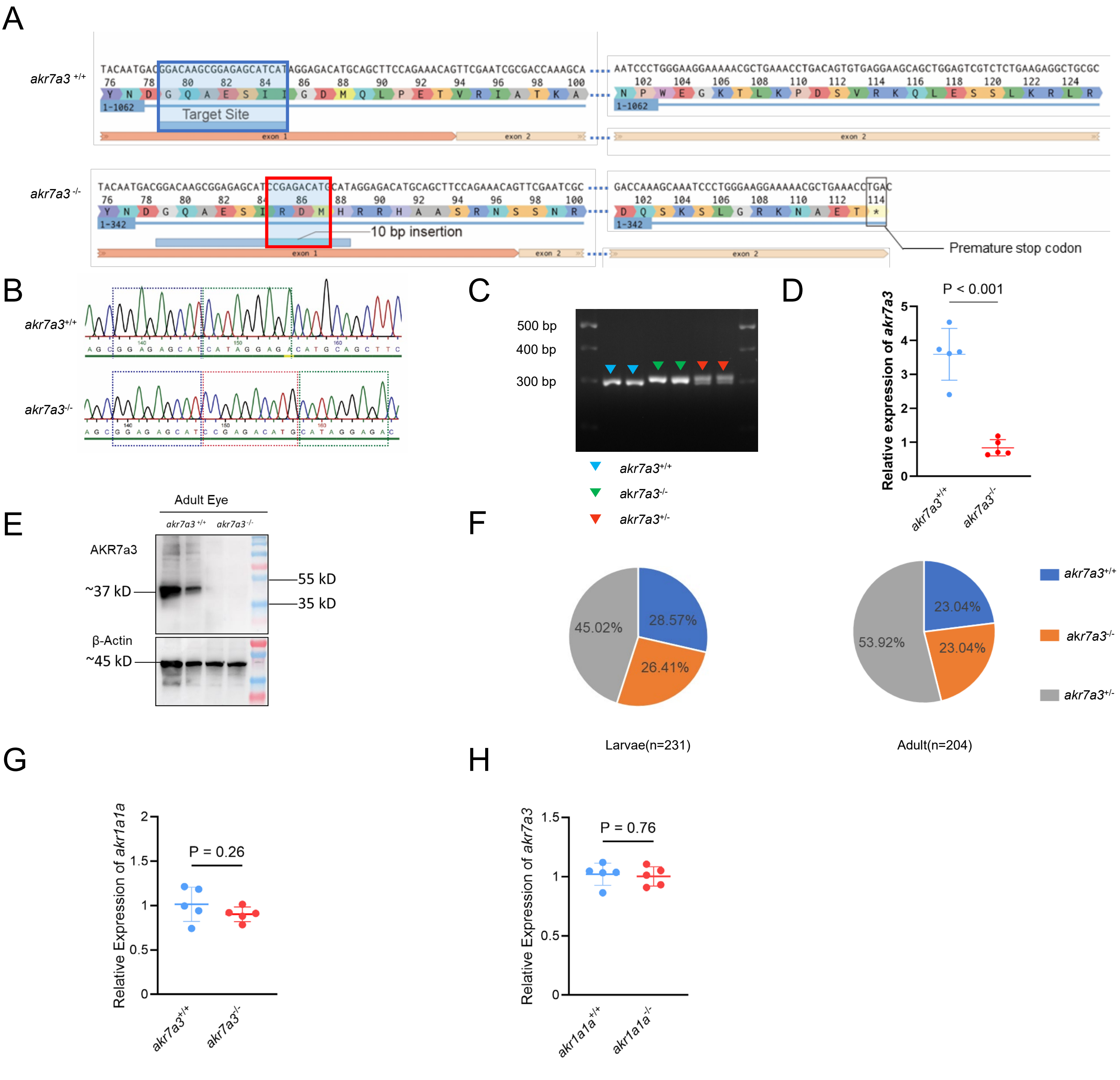

Figure S2. Generation and characterization of the *akr7a3*$^{-/-}$ zebrafish using CRISPR/Cas9 (A-B) The *akr7a3* knockout line was designed to target exon 1 and cDNA sequencing confirmed a 10 bp insertion was successfully produced with CRISPR. This insertion introduces an artificial stop codon, marked with a star. Bp, base pair. (C) Genotyping-PCR gel analysis allows for the differentiation of *akr7a3*$^{+/+}$, *akr7a3*$^{-/-}$, and *akr7a3*$^{+/-}$ zebrafish which were marked with blue, green, and orange arrows above respective band. (D) RT-qPCR analysis indicated *akr7a3* mRNA was significantly reduced in *akr7a3*$^{-/-}$ adult liver, n = 5. (E) Western blot analysis showed the loss of the AKR7a3 protein in *akr7a3*$^{-/-}$ adult eyes, n = 4. (F) Genotype distribution of larvae and adult zebrafish reproduced by heterozygous zebrafish, indicating the survival rate was not significantly changed between *akr7a3*$^{+/+}$ and *akr7a3*$^{-/-}$ zebrafish, n=231 and n=203. (G-H) 5 dpf old larvae from *akr7a3*$^{-/-}$ and *akr1a1a*$^{-/-}$ mutants were used to evaluate potential compensatory expression between *akr7a3* and *akr1a1a.* RT-qPCR analysis showed *akr7a3* knockout does not alter *akr1a1a* expression and vice versa. Each data point in this figure represented one adult fish or one clutch of larvae, with each clutch containing 37 to 50 larvae. The bars indicate values of mean ± SD. Statistical analysis was performed by Student's t-test.

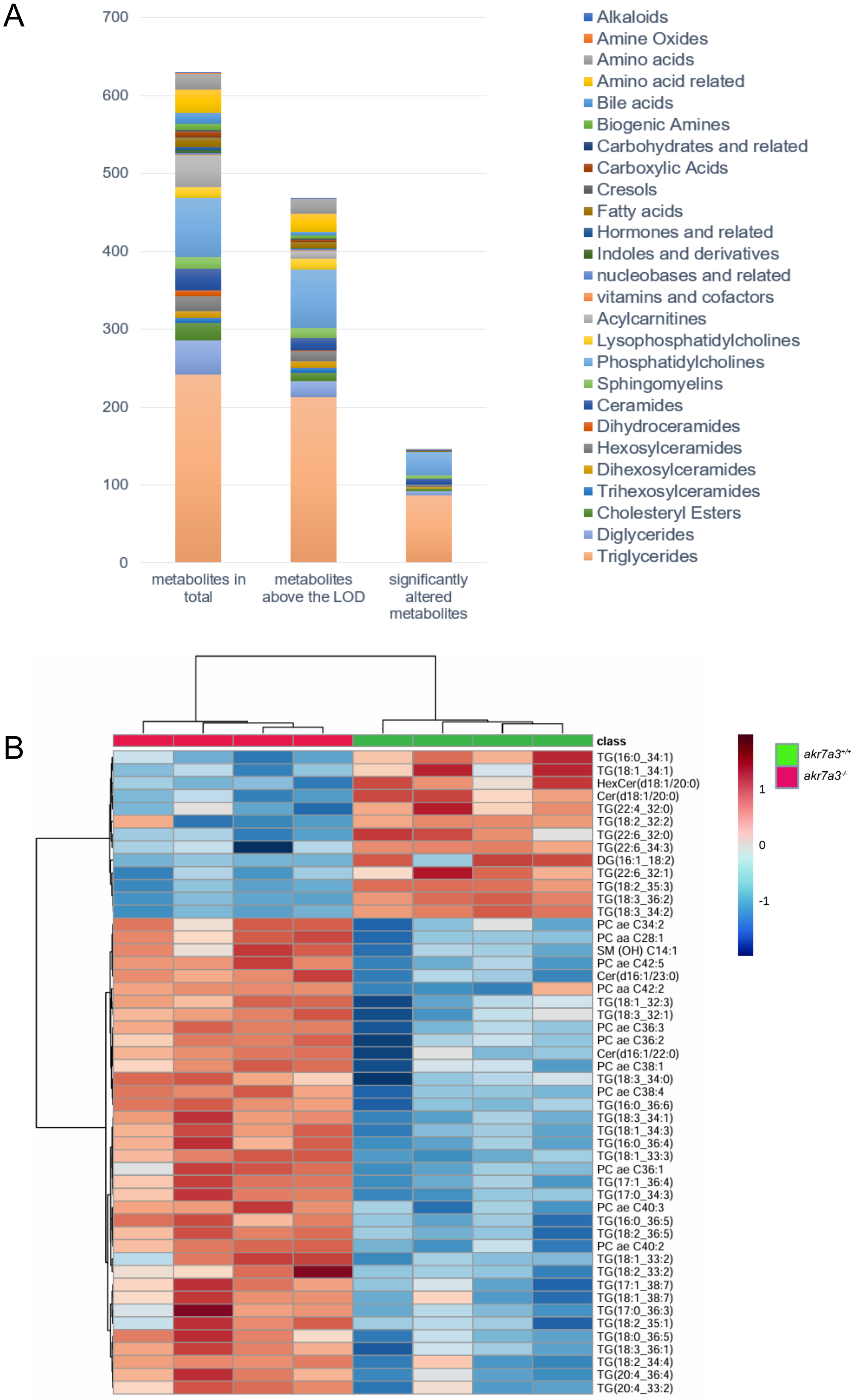

Figure S3. Metabolomic analysis of *akr7a3* zebrafish larvae at 96 hpf. (A) Overview of analyzed metabolites: 630 metabolites were analyzed; 469 metabolites were above LOD; 146 metabolites were significantly altered between *akr7a3*$^{+/+}$ and *akr7a3*$^{-/-}$ zebrafish larvae samples. (B) Heatmap based on metabolomic data indicated alterations of metabolites between *akr7a3*$^{-/-}$ and *akr7a3*$^{+/+}$ zebrafish larvae. n=4 clutches with 50 larvae. LOD, limit of detection.

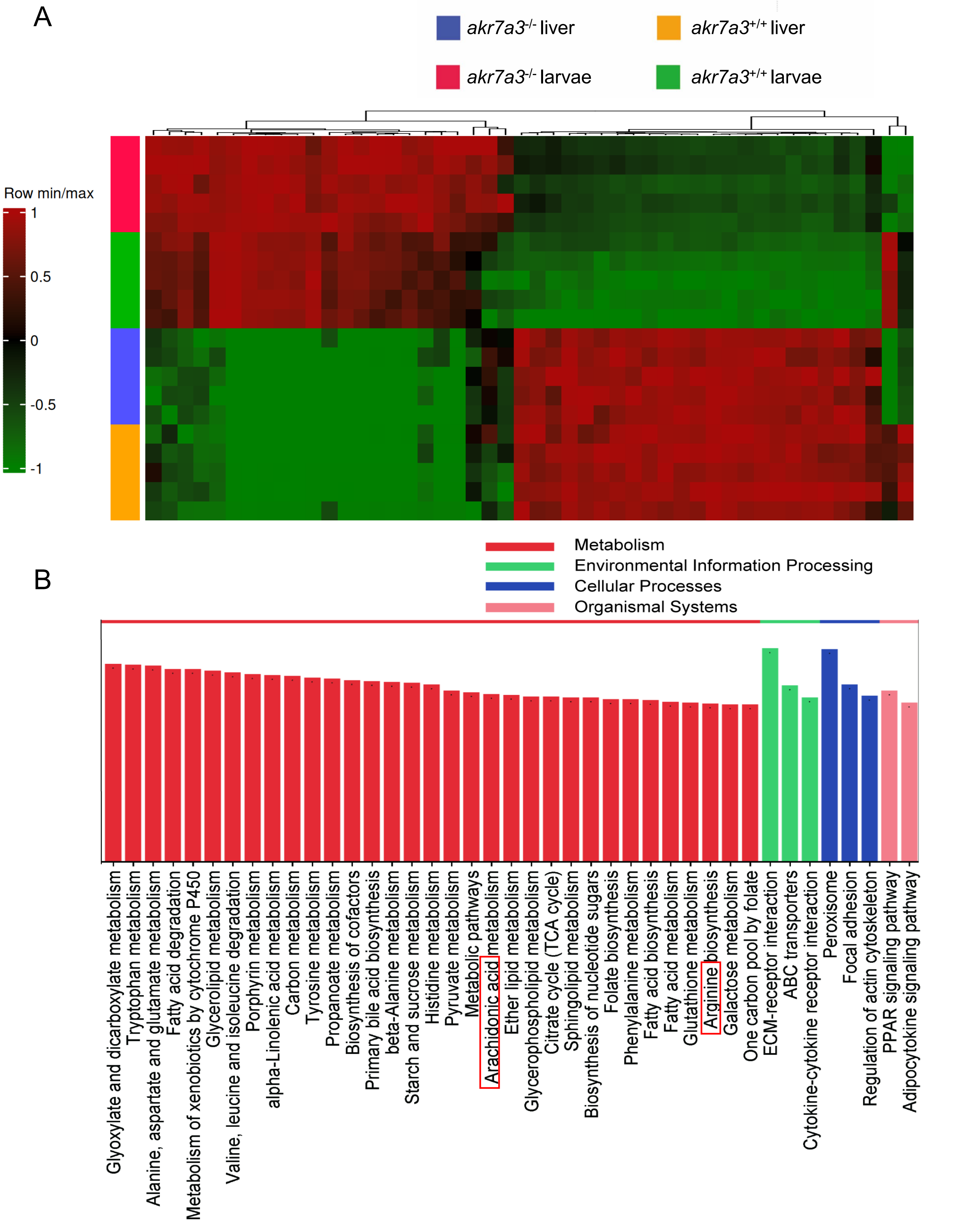

Figure S4. Transcriptomic analysis of *akr7a3* zebrafish larvae and adult. (A) Clustering analysis of RNA-seq disclosed that the gene expression profiles were significantly altered in larvae at 120hpf, and in adult livers between *akr7a3*$^{-/-}$ and *akr7a3*$^{+/+}$ zebrafish. (B) KEGG enrichment analysis of RNA-seq confirmed the alterations in metabolism, however the RNA-seq data also disclosed considerable changes in ECM-receptor interaction and peroxisome between *akr7a3*$^{-/-}$ and *akr7a3*$^{+/+}$ zebrafish. ECM, extracellular metrix. n = 5 clutches with 30 larvae or n = 5 adult livers.

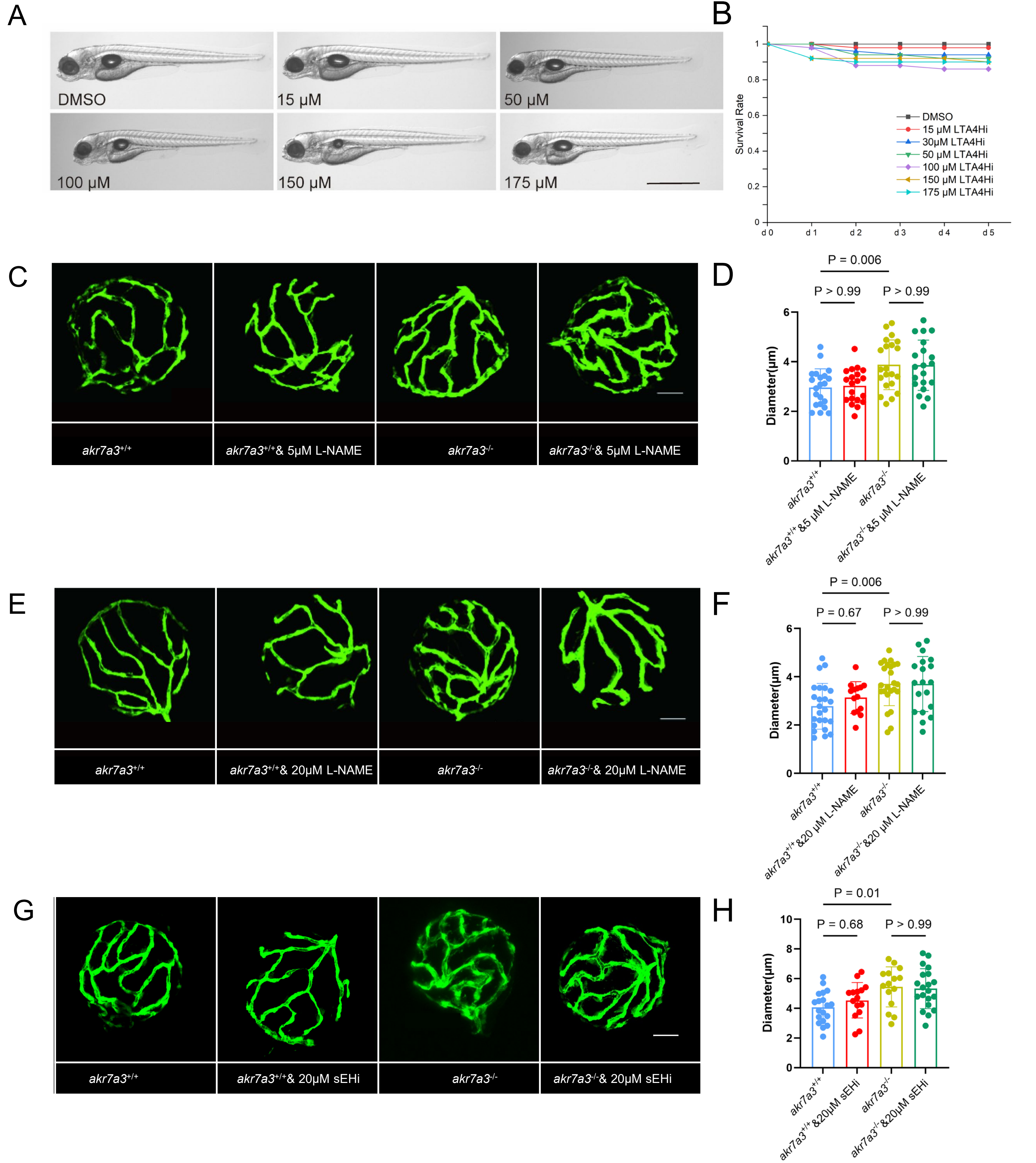

Figure S5. LTA4Hi toxicity test and hyaloid vasculature rescue experiment in larvae (A) Representative microscopic images of 96 hpf zebrafish larvae following treatment with 0-175 μM LTA4Hi. Scale bar: 200 μm. (B) Quantification of survival rates disclosed that even 175 μM LTA4Hi had limited effect on larvae survival and development, n = 50. (C-H) Representative confocal microscopy images and quantification of hyaloid vasculature alteration in *akr7a3*$^{+/+}$ and *akr7a3*$^{-/-}$ zebrafish larvae treated with 5 μM L-NAME, 20 μM L-NAME and 20 μM sEHi at 5 dpf. White scale bar = 30μm. The quantification indicated a significant increase in hyaloid vasculature diameter in *akr7a3*$^{-/-}$ larvae compared to *akr7a3*$^{+/+}$ zebrafish, but no rescue of diameter was observed after treatment with 5 μM L-NAME, 20 μM L-NAME or 20 μM sEHi, n = 18-24. One datapoint indicates one hyaloid per larva. The bars indicate mean±SD values. Statistical analysis was performed by one-way ANOVA. LTA4Hi, leukotriene A4 hydrolase inhibitor; sEHi, epoxide hydrolase inhibitor; L-NAME, N_{ω}-Nitro-L-Arginine Methyl Ester hydrochloride.

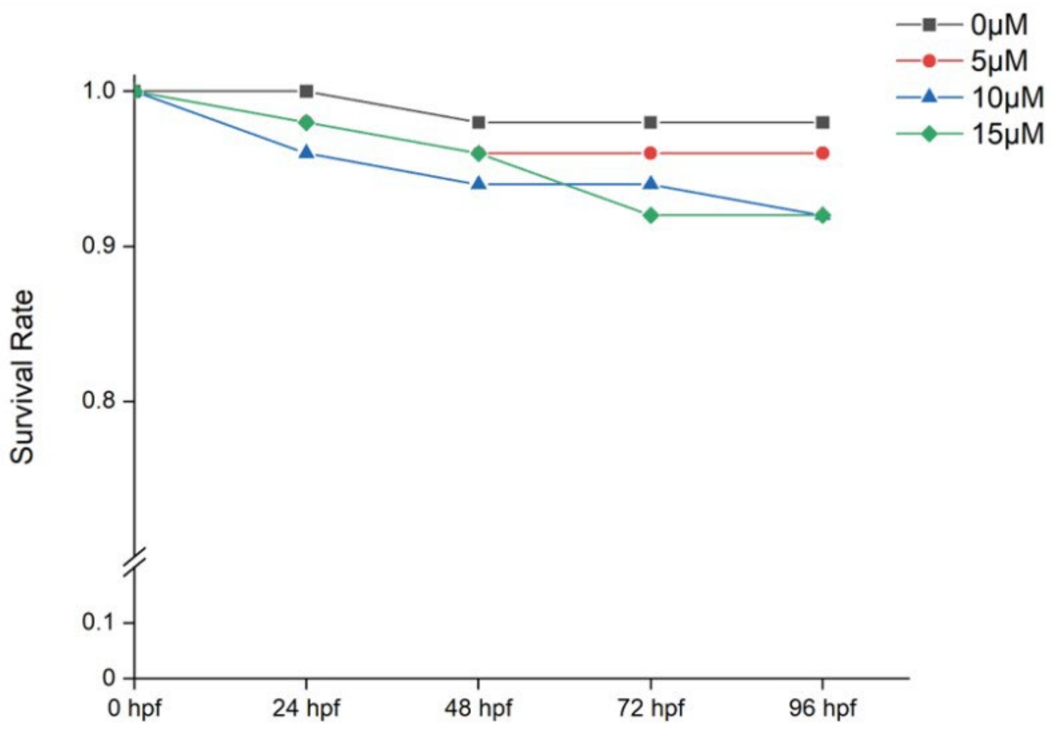

Figure S6. ACR toxicity assay in zebrafish larvae. (A) Survival rates decreased with increasing ACR concentrations in the larvae, indicating a potential dose-dependent effect of ACR toxicity within the tested range, n = 50.
