# Supplementary material for: Endogenous acrolein accumulation in akr7a3 mutants causes microvascular dysfunction due to increased arachidonic acid metabolism
Source: Redox Biol. 2025 Apr 17;83:103639. doi: 10.1016/j.redox.2025.103639 (PMC12051060; doi:10.1016/j.redox.2025.103639)
Supplement: Multimedia component 2 [file mmc2.pdf]

Primer Name	Sequence
akr7a3_CRISPER_forward	TAGGCCTGTAGTGTGTCCTGGA
akr7a3_CRISPER_reverse	AAACTCCAGGACACACTACAGG
akr7a3_genotyping_forward	TGACCGCACAGATGTCCACC
akr7a3_genotyping_reverse	GATCAGTCTCCCTACAGTAC
akr7a3_qPCR_forward	GTCACTCTGCTGGGCACCAT
akr7a3_qPCR_reverse	TTTGGTCGCGATTCGAACTGT
arnt2_qPCR_forward	AGCCAGACAGAGGTCTTCCA
arnt2_qPCR_reverse	CCGAGGTCAGCAAAGTCTTC
b2m_qPCR_forward	TGCCTTCACCCCAGAGAAAGGA
b2m_qPCR_reverse	CTCGCTGCCCGGTTTGGATTTA
LTA4H_qPCR_forward	CTCCAACTGTGTTGGCTGGA
LTA4H_qPCR_reverse	GAGCTGCTCACCCTCCATAC
COX-2_qPCR_forward	ACCAGGGCGTGTGTTTATCC
COX-2_qPCR_reverse	GTGAGAAGCTCAGGGGTAGTG
sEH_qPCR_forward	GGCTGTTCAAGTCAAGGACG
sEH_qPCR_reverse	TGGATCTTCACACCAGGCTTAATA
CYP2_qPCR_forward	ATTCCATTTTCCCTGGGGCTG
CYP2_qPCR_reverse	CCAAAGTGCTCTTCTGCGCT
AhR_qPCR_forward	GCCTGCGATGCAAAGGGTAA
AhR_qPCR_reverse	CCCATTTCCAACGGTTGTCC
Junb-a_qPCR_forward	GTCAGCTTCAGTACGTCCCA
Junb-a_qPCR_reverse	AAGTCGATTTCGGAGCGGAG
Junb-b_qPCR_forward	GGTCCTGAGGCACATGAACA
Junb-b_qPCR_reverse	TGAAAGTCCCGTCGCGTTTA
LTB4R_qPCR_forward	TGCCATTTTATCGCTCTGTGG
LTB4R_qPCR_reverse	AACAGGATGAAGGCGAAGGG
CysLT1R_qPCR_forward	AATCAACTTCGAAAGGGGCA
CysLT1R_qPCR_reverse	ACCACTCATGGAGGTCCTGT
CysLT2R_qPCR_forward	CAATACTCTGCCGGTTTGGC
CysLT2R_qPCR_reverse	ATGCATTCATGTCCGTTCCTTTT
CysLT3R_qPCR_forward	AGAAGATGACAGCTGCTGCG
CysLT3R_qPCR_reverse	GCAGTTCTGGCTTCTGTTGC
TNF-α_qPCR_forward	CCTGCTTGCAGATTGAGCGGAT
TNF-α_qPCR_reverse	AAGCTTGAGAGTCGGGCGCTT
IL-1β_qPCR_forward	CTCCGCTCCACATCTCGTA
IL-1β_qPCR_reverse	TCCTGTCCATCTCCACCATC
IL-6_qPCR_forward	CCTCAGTCCTGGTGAACGA
IL-6_qPCR_reverse	CACGCTGGAGAAGTTGAACA
M-CSF_qPCR_forward	CAACTAACCACCAGCACAGAG
M-CSF_qPCR_reverse	GAGGCAGTAGGCAGTGAGAA

Table continued

Primer Name	Sequence
akr1a1a_qPCR_forward	GGACCTCTACCTCATTCACTGG
akr1a1a_qPCR_reverse	CCCTTGGTCAACCAGCTTC
Nephrin_qPCR_forward	TTTTTGCTTCTTGTGGATTTCA
Nephrin_qPCR_reverse	ATTACGCGGCTCCGTCTT
Podocin_qPCR_forward	GGAGTTATTAGCATTATTGGAGACTGA
Podocin_qPCR_reverse	CAGCTCACAAACTCCAAGGTATT
